# Herpes Labialis Manifesting as Recurrent Erythema Multiforme and Solitary Ulcer on Face

**DOI:** 10.4103/0974-777X.52982

**Published:** 2009

**Authors:** Biju Vasudevan, Ashish Bahal, Vinod Raghav

**Affiliations:** *Department of Dermatology, MH Shillong, Shillong, Meghalaya - 793 001, India*

**Keywords:** Acyclovir, Erythema multiforme, Herpes labialis

## Abstract

A 65-year-old lady presented with recurrent crusting of lips and ulceration on face since the last three weeks. History revealed recurrent herpes labialis during the previous three years. Examination showed hemorrhagic crusting of lips and a solitary crusted ulcer on the right cheek. The patient showed partial improvement with antiviral therapy. On withdrawal of initial therapy, the patient developed classical lesions of herpes labialis and erythema multiforme. Investigations confirmed herpes simplex infection and erythema multiforme. All the lesions including the ulcer on the face responded to maintenance therapy with antivirals. Though herpes infection has been found to cause ulcers especially in the peri-anal region in immunosuppressed individuals, it is the first time in literature that a facial ulcer has been ascribed to herpes simplex virus that too in an immunocompetent individual.

A 65-year-old lady presented to the Dermatology OPD with complaints of crusting of lips and a large ulcer on the face of three weeks duration. There was a burning sensation over the ulcer on exposure to sunlight. The patient also gave history that she had three similar episodes of crusting of lips and ulceration on the same area of face during the last one year. On repeated probing, the patient gave history of episodic fluid-filled lesions around the mouth since the last 3 years. Dermatological examination revealed a solitary 8 × 6 cm superficial ulcer with overlying crusts on the right cheek [Figure 1]. The floor of the ulcer was erythematous and mildly tender. Hemorrhagic crusting of the lips was also present [Figure 2]. With a provisional diagnosis of recurrent EM due to HSV, the patient was prescribed oral Acyclovir 200 mg 5 times a day for 7 days and topical supportive therapy. Within 2 weeks, the hemorrhagic crusting had regressed and the lesion on the cheek had reduced in size. After 2 weeks of stopping treatment, the patient reported back with multiple grouped vesicles in the peri-oral region along with multiple oral erosions [Figure 3]. Careful examination of the body also revealed a solitary target lesion on the right thigh [Figure 4].

**Figure 1:**
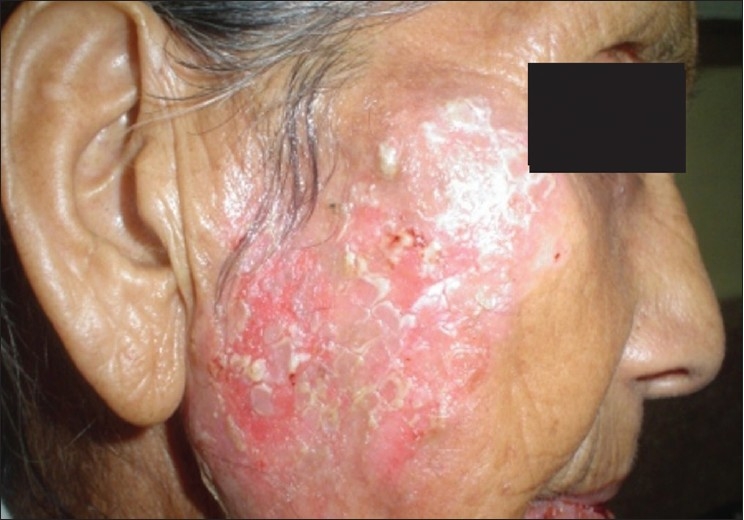
Solitary ulcer on face

**Figure 2:**
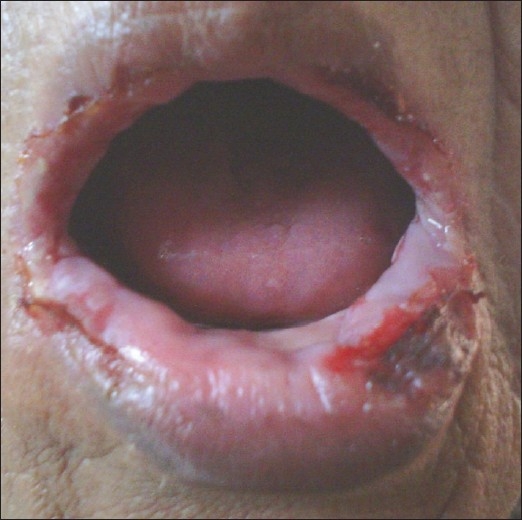
Hemorrhagic crusting of lips

**Figure 3:**
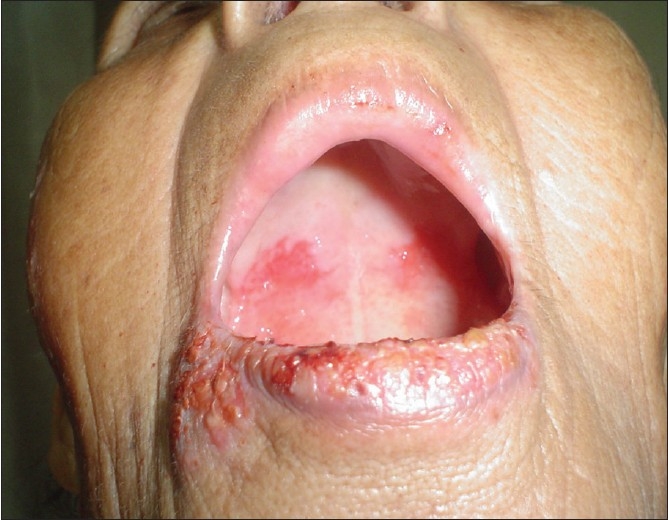
Herpes labialis with oral erosions

**Figure 4:**
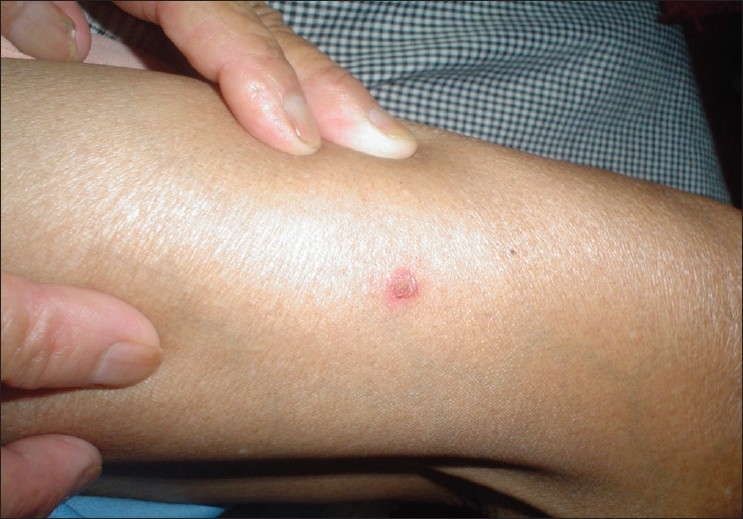
Classical target lesion of erythema multiforme

Tzanck smear from the peri-oral lesions and floor of the ulcer showed multinucleate giant cells. Histopathological examination of the solitary bulla on the right thigh showed features of classic erythema multiforme while that from the ulcer on the cheek confirmed viral etiology [Figure 5]. Anti-herpes simplex Type 1 IgG and IgM antibodies were both positive. ELISA for HIV and ANA were negative. CT scan of the chest and abdomen did not reveal any occult neoplasm. The patient was also given maintenance treatment with oral Acyclovir 400 mg twice daily for 6 months. The ulcer on the cheek completely regressed in 4 weeks [Figure 6]. There was no recurrence of any of the lesions after one year of follow up.

**Figure 5:**
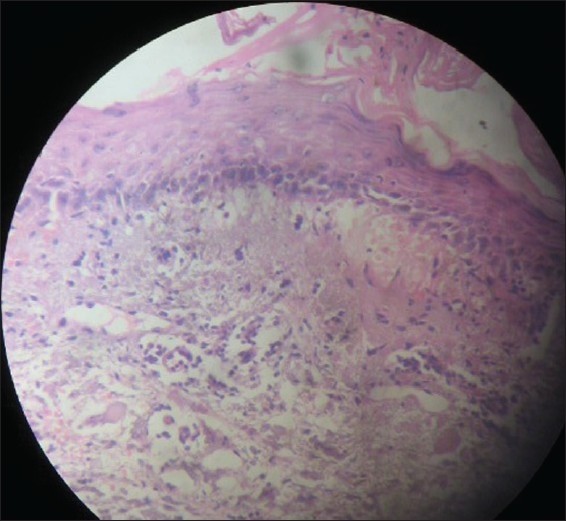
Biopsy from ulcer on face revealing features of herpetic ulcer

**Figure 6:**
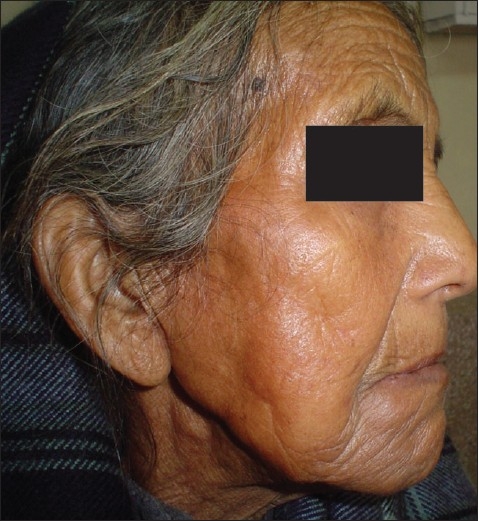
Fully healed lesions after treatment

Erythema multiforme is characterized by a polymorphous eruption composed of symmetrically distributed macules, papules, bullae and typical target lesions with a propensity for the distant extremities and the oral mucosae. Approximately 65% cases of recurrent EM give history of preceding herpes labialis.[1] Recurrent herpes associated EM (HAEM) can be precipitated by sun exposure.[2] HSV-specific T-cell response to the viral antigens is most likely involved in HAEM pathogenesis.[3] This condition is rare in persons younger than 3 years and older than 50 years. Thus our patient was a rare case of recurrent EM in older age.

Patients with >5 episodes/year, severe recurrences or unrecognizable prodromes of herpes simplex may be best managed with long-term suppressive antiviral prophylaxis.[4] Oral acyclovir 400 mg twice daily is effective in suppressing herpes labialis in immunocompetent adults confirmed to have frequently recurrent infection.[5] Newer drugs like famciclovir and valacyclovir are equally effective, if not better.[6]

Unusual cutaneous manifestations of herpes virus infection like peri-anal ulcers (HSV-2) and pyoderma gangrenosum-like lesions have been seen in immunocompromised patients.[7] Our patient, in addition to recurrent EM following herpes labialis, itself a rare condition in older patients, also had a crusted superficial ulcer on the face which responded to Acyclovir. Such an association with herpes infection has not been mentioned earlier in literature and this case is very unusual also for the fact that such a manifestation occurred in an immunocompetent individual infected with HSV.
